# A Model for Core Responses and Species-Specific Reprogramming to Whole-Genome Duplication in Plants

**DOI:** 10.3390/life16101643

**Published:** 2026-09-30

**Authors:** Yuyao Li, Jiaqing Yuan, Zhiyong Xiong, Kanglu Zhao

**Affiliations:** 1 State Key Laboratory for Development and Utilization of Forest Food Resources, Zhejiang A&F University, Hangzhou 311300, China; lyydeyouxiangya@163.com; 2 Key Laboratory of Herbage and Endemic Crop Biology, Ministry of Education, Inner Mongolia University, Hohhot 010070, China; 3 College of Ecology, Lanzhou University, Lanzhou 730000, China

**Keywords:** polyploid, whole-genome duplication, core responses, species-specific reprogramming, plant breeding

## Abstract

Whole-genome duplication (WGD) is a fundamental evolutionary force in plants, driving diversification and offering immense potential for crop improvement. While WGD’s molecular effects are well-studied in individual species, the consistency of these responses across diverse lineages remains largely unknown. Here, we integrated multi-omics datasets from established WGD events across 20 plant species, encompassing both experimentally induced and naturally occurring polyploids, to elucidate shared molecular adaptations to genome doubling. Our analysis identified 68 orthologous core WGD-responsive genes, predominantly involved in stress response, hormone signaling, and cellular homeostasis. Interestingly, despite considerable divergence in individual metabolites across species, their enriched pathways consistently converged on osmotic adjustment, redox regulation, and membrane remodeling. This convergent cascade of core transcriptional regulation and metabolic outputs thus defines a foundational adaptive program across diverse species. However, concurrently, extensive transcriptional and metabolic reprogramming exhibited strong species specificity. Based on this duality, we propose a novel model of core responses and species-specific reprogramming to WGD. This model provides crucial insights into polyploid stabilization and trait diversity, by highlighting an endogenously activated stress-buffering state that is pivotal for WGD establishment. Moreover, the identified core genes offer promising molecular markers for targeted polyploid breeding strategies.

## 1. Introduction

Whole-genome duplication (WGD), or polyploidization, is a recurrent genetic event in plant evolution and breeding [1]. It is widespread across plants, especially angiosperms, where roughly 30–50% of extant species are estimated to be recent polyploids, and nearly all major lineages retain signatures of ancient polyploidy [2,3]. Unlike single-gene mutations, WGD duplicates entire chromosome sets at once, thereby rapidly reconfiguring genome structure, gene dosage relationships, and regulatory networks [4,5]. For this reason, WGD is regarded as a macroevolutionary process with major evolutionary and applied significance [6,7]. It serves as a critical genetic basis for trait innovation, environmental adaptation, and speciation, simultaneously offering immense potential for crop domestication, yield enhancement, stress tolerance breeding, and bioeconomy-related applications [8,9,10,11]. For example, tetraploid *Arabidopsis thaliana* shows characteristic organ enlargement [12,13], while experimentally induced polyploid rice shows altered plant morphology, fertility, and yield-related traits [14,15]. Additionally, polyploid citrus demonstrates enhanced drought tolerance [16]. Thus, elucidating the molecular responses and trait reshaping associated with WGD is paramount for both understanding plant evolutionary mechanisms and guiding effective polyploid breeding.

At the evolutionary scale, ancient WGD events have profoundly impacted plant genome structure, duplicate gene retention/loss, and lineage diversification [6,17]. Remarkably, recent macroevolutionary studies indicate that ancient polyploidization events are not randomly distributed through time [10]. Instead, they are significantly enriched during major episodes of environmental disruption, including oceanic anoxic events, the Cretaceous–Paleogene extinction, the Paleocene–Eocene Thermal Maximum, the Eocene–Oligocene transition, and the Miocene climatic transition [6,18]. These patterns suggest a strong link between polyploidization and lineage persistence as well as subsequent diversification under extreme environmental conditions [19]. However, inferring and dating ancient WGD events typically relies on indirect evidence, such as synteny analyses, Ks distributions, and phylogenetic reconstruction. These inferences can be affected by gene loss, biased retention, and the enrichment of dosage-sensitive genes [20,21]. Consequently, caution is warranted when inferring the biological consequences of WGD from such paleogenomic signals. Specifically, it remains challenging to disentangle the effects of WGD from secondary outcomes molded by long-term diploidization [19,22].

To overcome these challenges, recently established polyploids provide valuable systems for investigating the core molecular responses to WGD [23]. Previous studies using such systems have shown that WGD can substantially affect organ morphology, stress tolerance, and both transcriptomic and metabolic states [24,25]. However, analyses in individual species, such as transcriptomic and metabolomic studies of synthetic *Solanum* autotetraploids, have often reported molecular perturbations to be largely stochastic or genotype-specific, with only limited evidence for broadly transcriptional and metabolic signals across investigated species [26]. This has led to ambiguity regarding whether observed responses represent general consequences of WGD or merely lineage-specific outcomes, and consequently, a fundamental understanding of the shared adaptive mechanisms across diverse plant lineages remains elusive.

Therefore, to address these knowledge gaps and move beyond species-specific observations, we integrated multi-omics datasets from 20 diverse plant species after established WGD events, drawing from both experimentally induced and naturally occurring polyploids. Employing a comprehensive cross-species comparative framework, we aimed to identify generalizable features of the core molecular response to WGD and their underlying functional modules. Specifically, utilizing unified ortholog mapping and comprehensive enrichment analyses, we evaluated the extent of commonality and divergence across genes and pathways. Ultimately, this study proposes a novel model that elucidates how WGD orchestrates molecular responses and drives trait innovation, with implications for polyploid breeding.

## 2. Materials and Methods

### 2.1. Data Collection

To investigate the molecular responses to whole-genome duplication (WGD) across diverse plants, a comprehensive multi-omics dataset was compiled, encompassing genome, transcriptome, and non-targeted metabolome profiles. This dataset included 20 angiosperm species, selected from publicly available databases and published literature (Supplementary Table S1). We specifically focused on collecting transcriptomic and metabolomic changes associated with established WGD events, comprising both experimentally induced and naturally occurring polyploids.

To minimize the influence of species-specific idiosyncrasies or physiological responses linked to polyploidization induction methods, we intentionally employed an extensive multi-species approach and prioritized the inclusion of data from mature polyploid lines that had undergone multiple generations of stable propagation or field cultivation. Crucially, this selection strategy allowed us to capture stable molecular and physiological signatures that have emerged over a relatively short evolutionary timescale following WGD. These lines thus represent a “core response” phase that is post-stabilization but still considered early relative to geological timescales and the protracted process of complete diploidization.

### 2.2. Phylogenetic Analysis

To illustrate the phylogenetic relationships among the selected species, a species tree was constructed using the R package V.PhyloMaker2 [27], with the GBOTB.extended.TPL.tre mega-tree as a backbone. This method operates by grafting species onto the pre-existing backbone tree, thereby not requiring genomic sequence data. The resulting tree was subsequently visualized and annotated using tvBOT v2.6.1 [28].

### 2.3. Transcriptome Data Processing and Differential Expression Analysis

Raw RNA-seq reads were initially processed using fastp v0.23.2 [29] for quality control and adapter trimming with default parameters. Clean reads were then quantified against the corresponding species-specific reference genome and annotation using Salmon v1.10.3 [30], yielding read counts and transcript abundance estimates.

Differential expression analysis between WGD and control samples was performed using the DESeq2 R package [31]. Genes with an adjusted *p* value (*P*_adj_) < 0.05 and an absolute log_2_fold change (|log_2_FC|) > 1 were defined as differentially expressed genes (DEGs). For species with transcriptomic data from multiple studies/tissues, the union of DEGs identified across studies/tissues constituted the final DEG set for that species, a strategy employed to maximize the breadth of potential WGD responses captured per species. Crucially, we implemented a filtering step to ensure that each species contributed a set of DEGs with a minimum threshold of 1000 DEGs. This selection criterion guarantees a rich initial gene pool, encompassing a substantial number of genes capable of reflecting both shared and species-specific responses, thereby providing a comprehensive foundation for subsequent cross-species comparative analyses. To explore potential relationships between WGD-responsive DEGs and genomic attributes, Spearman’s rank correlation coefficient was calculated between the number of identified DEGs for each species and its genomic features.

### 2.4. Orthology Inference and A. thaliana Reference Mapping

To enable cross-species comparison of gene expression, protein sequences from all species included in the transcriptome analysis were clustered into orthogroups using OrthoFinder v3 [32]. This approach identifies sets of genes descended from a single gene in the common ancestor, thereby capturing various gene homology relationships including one-to-one, one-to-many, and many-to-many orthologs and paralogs that arise through speciation and gene duplication events. High-confidence orthologous groups, defined as those detected in at least 50% of the analyzed species, were retained for downstream analysis. For the purpose of identifying the most fundamental and shared transcriptional responses to WGD across diverse angiosperms, we employed a targeted mapping strategy. Specifically, we mapped DEGs from each species to their corresponding *A. thaliana* orthologs identified within these orthogroups.

*A. thaliana* was selected as the reference due to its extensive functional annotations and status as a central model organism, which greatly facilitates deeper functional interpretation. We acknowledge that *A. thaliana* is a derived diploid with a relatively streamlined genome, meaning this framework may not capture all species-specific gene families. However, this characteristic was leveraged as a stringent filter: our primary objective was to pinpoint the deepest evolutionary similarities that manifest even in a divergent model like *A. thaliana*. This approach, while potentially enriching for deeply shared, fundamental processes (including those broadly considered “housekeeping” or stress-responsive), aligns with our aim to uncover the core, ubiquitous transcriptional machinery critically involved in adapting to WGD across a broad phylogenetic range.

### 2.5. Identification of Core Transcriptional Responses

The stringent criterion for identifying core WGD-responsive genes was that these orthologs must be identified as differentially expressed in all analyzed species, including *A. thaliana*. Within this core set, we specifically chose not to distinguish between up- and down-regulated genes. This methodological decision was driven by the recognition that the precise direction of gene regulation (up- or down-regulation) is highly context-dependent across diverse species, tissues, and experimental conditions. Consequently, our primary objective was not to identify genes with a uniform directional change, but rather to identify genes consistently perturbed by WGD, considering them integral components of a coordinated, multi-directional molecular network crucial for adaptation to polyploidy. Functional enrichment analyses, including Gene Ontology (GO) and Kyoto Encyclopedia of Genes and Genomes (KEGG) enrichment, were conducted for the shared DEGs using TBtools-II v2.490 [33].

To mitigate inherent limitations of *A. thaliana*-centric ortholog mapping, including potential ID conversion inaccuracies, we employed a complementary, ortholog-independent strategy for assessing functional commonality. Specifically, GO enrichment analysis was performed independently for each species, using its full gene set as the background, to identify all significantly enriched GO terms. Species yielding more than 300 enriched GO terms were then included in a subsequent intersection analysis. Shared GO terms identified from this analysis were retained for downstream comparison and functional classification.

### 2.6. Cross-Species Metabolome Analysis

Orthogonal projections to latent-structures discriminant analysis (OPLS-DA) was applied using the R package Ropls. Metabolites exhibiting a *p* < 0.05 and a variable importance in projection (VIP) > 1 were defined as differentially accumulated metabolites (DAMs) within each respective species. Recognizing the inherent technical variability and analytical diversity across independently generated non-targeted metabolomics datasets—which involve distinct extraction protocols, LC/GC-MS platforms, chromatographic columns, and annotation confidence levels—we employed a multi-tiered analytical strategy for cross-species comparison. To facilitate consistent analysis, metabolite identifiers were standardized, and their corresponding metabolic pathways were annotated using MetaboAnalyst 5.0 (https://www.metaboanalyst.ca/, accessed on 15 June 2026). Subsequently, an intersection analysis was performed to identify DAMs and pathways shared across species.

### 2.7. Independent Validation Using Potato Transcriptome Data

To independently validate our findings, previously generated RNA-seq data from wild diploid potato and its derived autotetraploid [34] were utilized. Crucially, this autotetraploid material is a mature polyploid that has undergone multiple generations of propagation and field cultivation. This long-term stability precludes any confounding effects from initial chemical induction (e.g., colchicine) on its transcriptome profile. Following the methods described above, DEGs were identified, and GO enrichment analysis was performed using the *Solanum chacoense* genome (https://spuddb.uga.edu/download.shtml, accessed on 15 June 2026) as the reference. Subsequently, intersection analysis was conducted between the identified potato DEGs and GO terms, respectively, and the cross-species shared DEGs and GO terms. The statistical significance of these overlaps was assessed using a one-tailed Fisher’s exact test.

## 3. Results

### 3.1. Construction of a Cross-Species Dataset for Established WGD and Global Transcriptional Response Profiles

**Figure 1:**
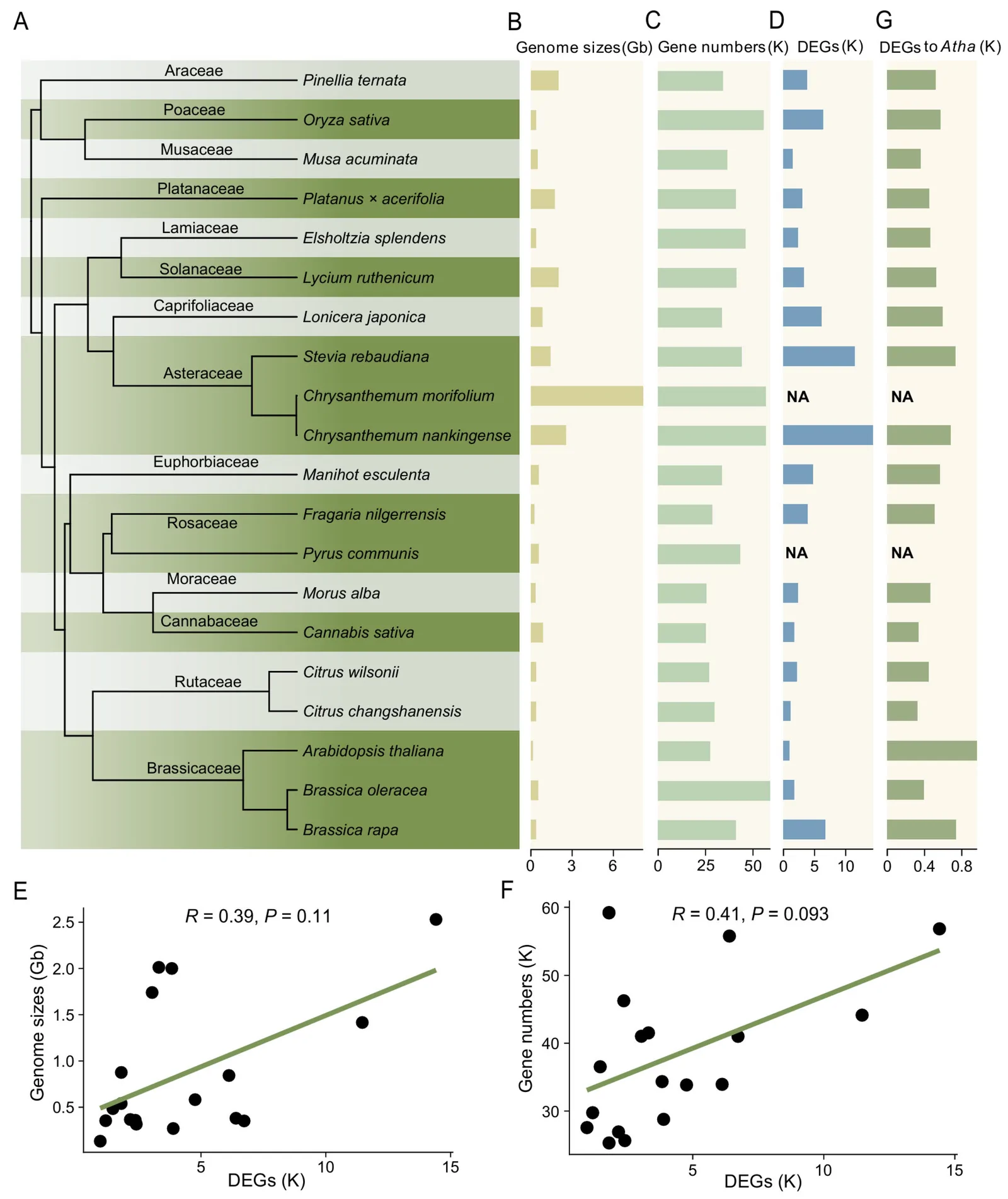
Phylogenetic relationships, genomic characteristics, and WGD-responsive DEGs across species. (**A**) A phylogenetic tree illustrating the evolutionary relationships and taxonomic distribution of the 20 species. (**B**) Genome sizes (Gigabases, Gb) for each species. (**C**) Total gene numbers (thousands, K) per species. (**D**) Numbers of differentially expressed genes (DEGs, K) identified in each species in response to WGD. (**E**,**F**) Scatter plots showing the correlations of the number of DEGs with genome sizes (**E**) and total gene numbers (**F**) across species. For both panels, Spearman’s rank correlation coefficient (*R*) and corresponding *p*-value are indicated. (**G**) Numbers of WGD-responsive DEGs from each species that were mapped to the *Arabidopsis thaliana* ortholog framework, facilitating cross-species gene-level comparison. “NA” indicates that transcriptomic data were not available for differential expression analysis in these species, although metabolomic data might have been collected.

To systematically characterize the core molecular response to whole-genome duplication (WGD) in plants, we integrated transcriptomic and metabolomic datasets from established WGD events across multiple taxa. In total, our comprehensive dataset comprised 20 plant species (Supplementary Table S1). The selected species span major angiosperm lineages and represent 14 families (Figure 1A), thus providing extensive phylogenetic coverage suitable for identifying cross-species molecular responses to WGD. These species also exhibited substantial variation in genomic background: genome sizes ranged from 0.13 to 8.15 Gb (Figure 1B), and gene numbers ranged from 25 to 59 K (Figure 1C). This broad range underscores the genomic heterogeneity captured in our dataset.

We then identified differentially expressed genes (DEGs) before and after WGD in each species (Figure 1D; Supplementary Table S2). These results indicated broad transcriptome-wide reprogramming triggered by WGD, with the number of DEGs varying substantially across species. Investigating whether species-specific DEG numbers correlated with genomic attributes, our association analysis revealed no statistically significant correlation between the number of DEGs and genome size (Figure 1E; *p* = 0.11) or total gene number (Figure 1F; *p* = 0.093), consistent with observations in previous studies [35,36,37]. Despite this variability, to identify potential core response genes and functional modules across these diverse species, we subsequently mapped the DEGs to an *Arabidopsis thaliana* ortholog framework for cross-species comparison (Figure 1G; Methods).

### 3.2. Shared DEGs Across Species Reveal Common Stress and Transcriptional Regulation Responses After WGD

**Figure 2:**
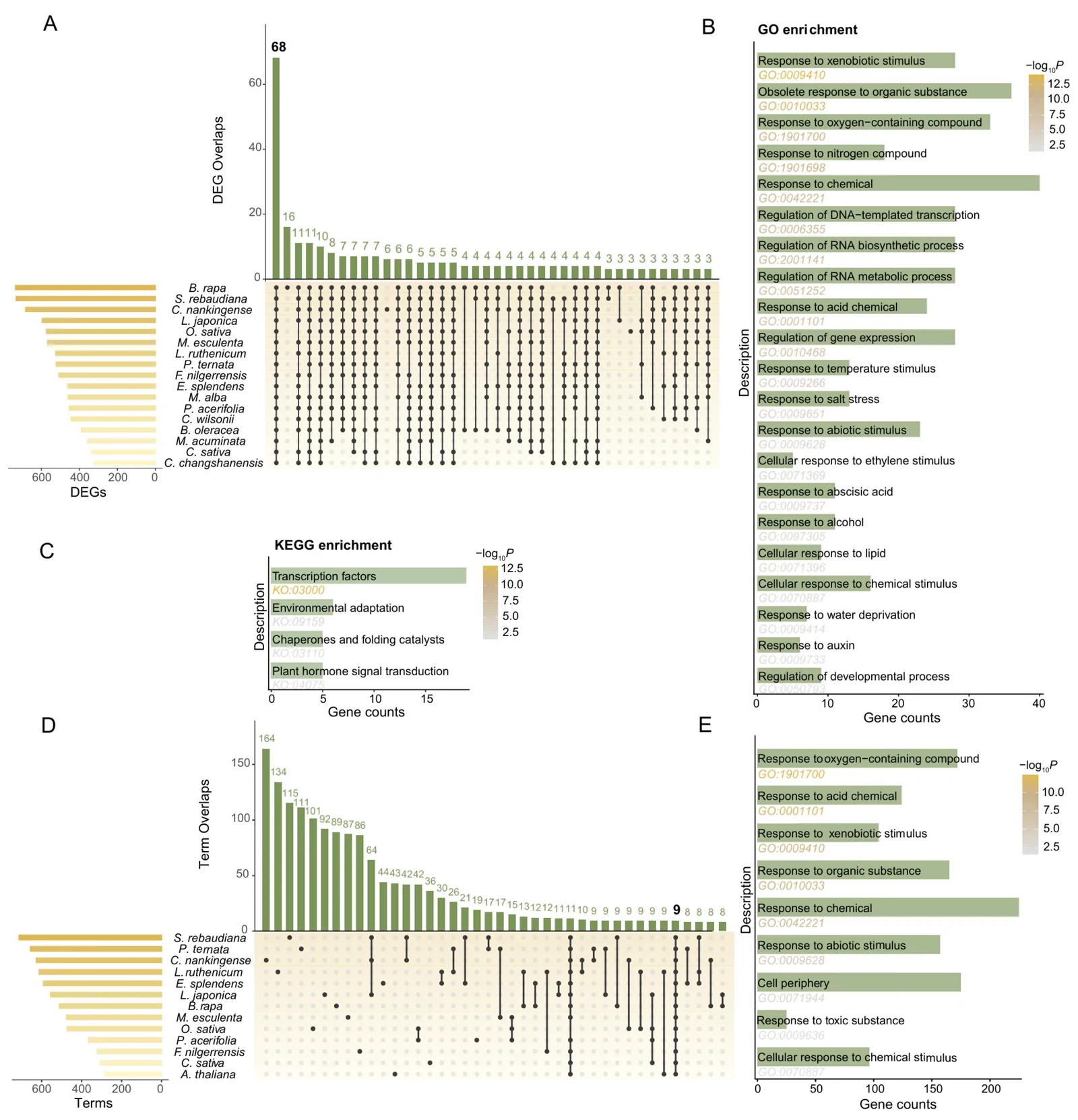
Cross-species identification and functional characterization of core WGD-responsive genes. (**A**) UpSet plot illustrating the overlap of DEGs across species. The left bar plot displays the total number of DEGs identified in each species. The main UpSet plot visualizes the shared DEGs among species, where vertical bars indicate the number of DEGs common to the connected species. (**B**,**C**) Gene Ontology (GO; (**B**)) and Kyoto Encyclopedia of Genes and Genomes (KEGG; (**C**)) enrichment analysis of the 68 identified core DEGs. (**D**) UpSet plot illustrating the overlap of significantly enriched GO terms across species. The left bar plot displays the total number of enriched GO terms for each species. The main UpSet plot visualizes the shared enriched GO terms among species, where vertical bars indicate the number of terms common to the connected species. (**E**) Functional enrichment of shared GO terms. Horizontal bars represent the number of genes (Gene counts) associated with each enriched term, and the color intensity reflects the statistical significance.

To identify shared components of WGD-induced transcriptional reprogramming, we analyzed DEG overlap across species using the *Arabidopsis* ortholog framework (Methods). Our UpSet analysis revealed that while DEG composition varied substantially among species, a core set of 68 DEGs was consistently shared across all 18 transcriptome-analyzed species (Figure 2A; Supplementary Table S3). Gene Ontology (GO) enrichment analysis of these 68 shared DEGs indicated that they were mainly associated with two broad categories: stress response and regulatory processes (Figure 2B; Supplementary Table S4). Specifically, stress-related terms encompassed responses to salt stress, water deprivation, temperature, and various chemical stimuli. Concurrently, regulatory terms largely pertained to hormone signaling, especially ethylene, abscisic acid, and auxin, alongside processes like transcription from DNA templates, RNA metabolism, and gene expression regulation. Collectively, these findings highlight that the shared DEGs are predominantly involved in stress sensing and subsequent regulatory reprogramming following WGD, functionally converging on abiotic stress responses, chemical stimuli responses, and the dynamic remodeling of expression programs.

KEGG enrichment analysis further corroborated this pattern (Figure 2C; Supplementary Table S5). The shared DEGs were significantly enriched in pathways related to environmental adaptation, plant hormone signal transduction, transcription factors, and chaperones and folding catalysts. These KEGG pathways align well with the GO results: environmental adaptation resonates with abiotic stress-related GO terms; hormone signaling and transcription factors underscore hormonal and transcriptional regulation; and chaperones and folding catalysts point to adjustments in protein homeostasis after WGD. Taken together, these findings strongly indicate that the shared cross-species transcriptional response to WGD involves a coordinated cascade encompassing abiotic and chemical stress responses, hormone-mediated regulation, extensive transcriptional reprogramming, and the maintenance of protein homeostasis.

### 3.3. GO Term Overlap Across Species Reinforces Common Functional Features After WGD

Acknowledging limitations of *A. thaliana*-centric ortholog mapping, we utilized a complementary, ortholog-independent approach for functional commonality (Methods). Specifically, we conducted individual GO enrichment analyses for the DEGs of each species. For the subsequent term-level overlap analysis (Figure 2D), we focused on species exhibiting over 300 enriched GO terms, additionally including *A. thaliana*. While the number of enriched terms varied across species, we nevertheless identified a consistent set of overlapping GO terms. This finding underscores that WGD-induced transcriptional reprogramming exhibits commonality not only at the gene level but also at the functional level.

These shared GO terms were primarily associated with abiotic stress and chemical stimuli responses (Figure 2E; Supplementary Table S6). Prominently featured terms included responses to oxygen-containing compounds, acid chemicals, and various organic and toxic substances. These findings suggest that diverse plant species commonly activate functional modules pertaining to environmental and chemical stress in response to WGD. Crucially, the term-level overlap was highly consistent with the enrichment results derived from the shared DEGs, with both analyses converging on abiotic stress and chemical response programs after WGD.

### 3.4. WGD-Induced Metabolic Changes Are Highly Species-Specific

**Figure 3:**
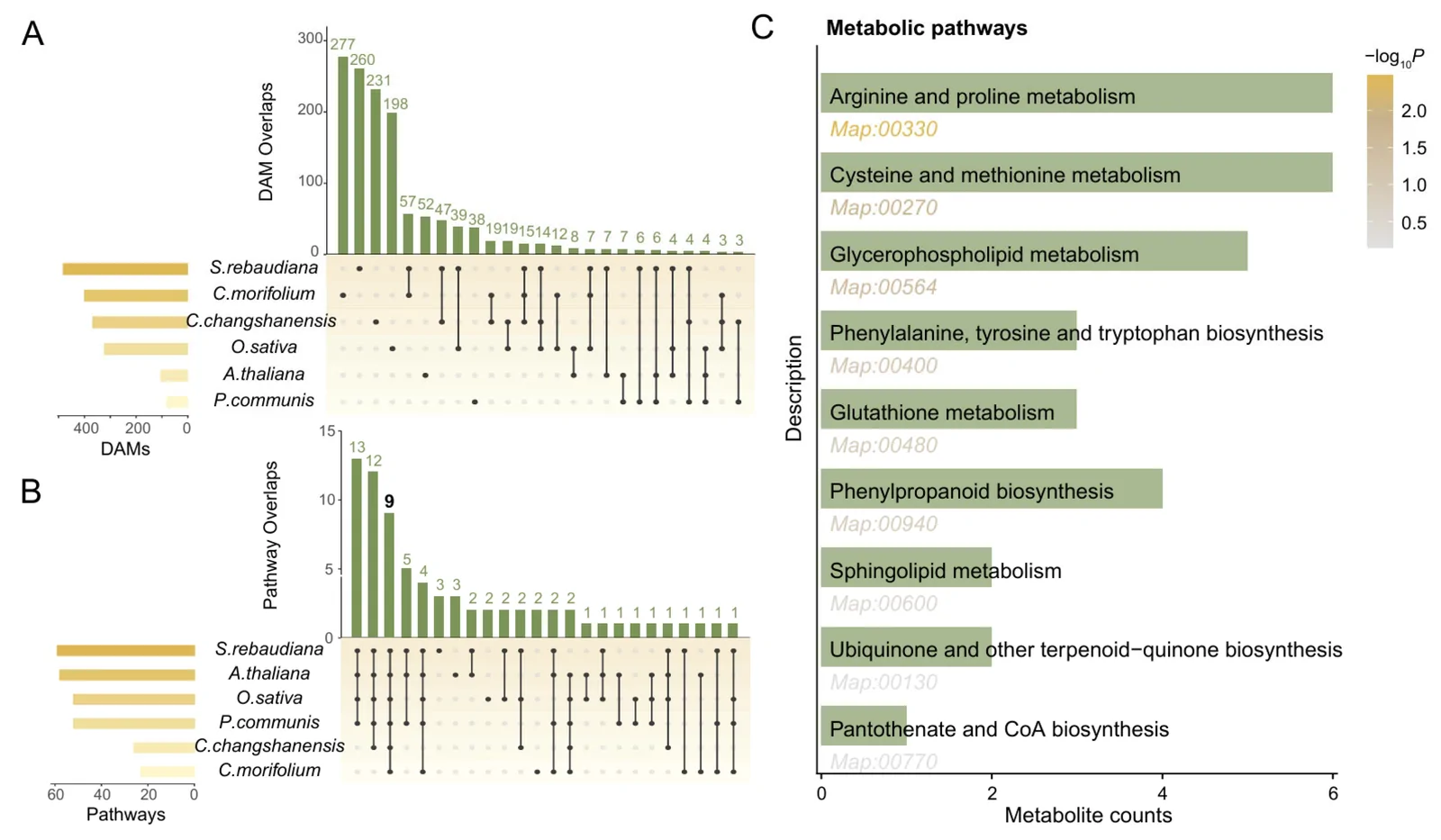
Cross-species analysis of differentially accumulated metabolites (DAMs) and enriched metabolic pathways. (**A**) UpSet plot illustrating the overlap of DAMs across species. The left bar plot displays the total number of DAMs identified in each species. The main UpSet plot visualizes the shared DAMs among species, where vertical bars indicate the number of DAMs common to the connected species. (**B**) UpSet plot illustrating the overlap of enriched metabolic pathways across species. The left bar plot displays the total number of enriched pathways identified in each species. The main UpSet plot visualizes the shared enriched pathways among species, where vertical bars indicate the number of pathways common to the connected species. (**C**) Functional enrichment of the shared metabolic pathways. Horizontal bars represent the number of metabolites (Metabolite counts) associated with each enriched pathway, and the color intensity reflects the statistical significance.

To investigate the physiological manifestations of genome doubling beyond the transcriptional level, we integrated non-targeted metabolomic data to assess downstream metabolic consequences of WGD. We first quantified the number of differentially accumulated metabolites (DAMs) in each species to gauge the overall intensity of the metabolic response (Methods). Results revealed that the scale of metabolic shifts demonstrated substantial variation among species, likely attributable to their diverse baseline metabolic architectures. For instance, *Stevia rebaudiana*, *Chrysanthemum morifolium*, *Citrus changshanensis*, and *Oryza sativa* exhibited a robust response with over 300 DAMs, whereas *A. thaliana* and *Pyrus communis* showed more subtle changes, with only approximately 100 DAMs identified in each (Figure 3A). These observations suggest that the magnitude of metabolic remodeling following WGD is highly taxon-dependent and heterogenous.

Cross-species overlap analysis further demonstrated that, even within a unified metabolite annotation framework, no single DAM was consistently shared by all analyzed species (Figure 3A). Species-specific DAMs were predominant, with the vast majority of metabolites being unique to individual species; at a maximum, only three species shared any identical DAMs. This pronounced idiosyncrasy contrasts sharply with our transcriptomic results, where shared DEGs remained discernible across 18 species. Consequently, the response to WGD appears to be more dispersed and species-dependent at the level of individual metabolites than at the level of transcripts.

### 3.5. Metabolic Responses to WGD Converge at the Pathway Level

Although no single DAM was shared across all species, DAMs in each species were assigned to metabolic pathways for cross-species comparison (Methods). The number of enriched pathways varied markedly among species, with *S. rebaudiana*, *A. thaliana*, *O. sativa*, and *P. communis* showing substantially more enriched pathways than *C. changshanensis* and *C. morifolium* (Figure 3B). Overlap analysis further showed that DAMs from different species consistently converged on nine shared metabolic pathways. This result indicates that, while WGD-induced metabolic responses are highly divergent at the level of individual metabolites, they show clear convergence at the level of pathways.

Further examination of these nine convergent metabolic pathways (Figure 3C; Supplementary Table S7) strongly corroborated the transcriptomic findings. Specifically, enriched pathways like arginine and proline metabolism, cysteine and methionine metabolism, and phenylpropanoid biosynthesis directly underpin abiotic stress response by producing osmolytes, antioxidants, and defensive compounds. Concurrently, altered glutathione metabolism and ubiquinone biosynthesis highlighted enhanced redox homeostasis and detoxification, key components of stress adaptation. Furthermore, dynamic changes in glycerophospholipid and sphingolipid metabolism indicated cellular and membrane remodeling, crucial for maintaining protein homeostasis and overall cellular function in the post-WGD context. Collectively, this strong convergence across transcriptomic and metabolomic data confirms that cross-species commonality after WGD primarily operates at the level of these core functional modules and overarching physiological adaptations.

### 3.6. Independent Validation of Core WGD-Induced Genes and Functions Using Autotetraploid Potato Transcriptomics

**Figure 4:**
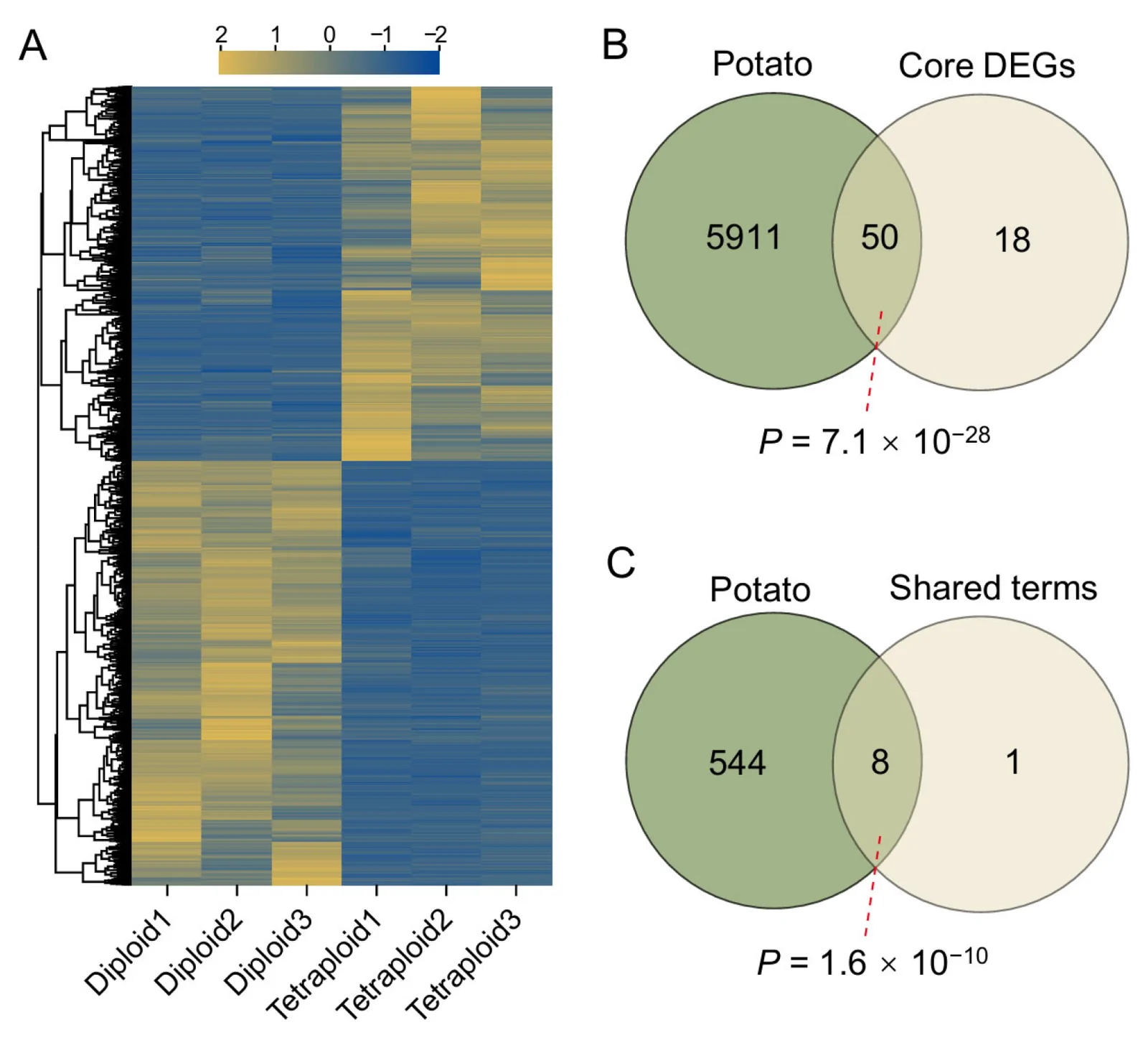
Validation of core WGD-responsive genes and pathways using potato transcriptomic data. (**A**) Heatmap visualization of DEGs in diploid (Diploid1–3) versus autotetraploid (Tetraploid1–3) potato. Each row represents a DEG, and columns represent biological replicates. The color scale indicates normalized gene expression levels, with yellow representing higher expression and blue representing lower expression. Hierarchical clustering is applied to genes. (**B**) Venn diagram showing the overlap between DEGs identified in potato (Potato) and the cross-species shared WGD-responsive DEGs (Core DEGs). (**C**) Venn diagram showing the overlap between significantly enriched GO terms identified in potato (Potato) and the cross-species shared WGD-responsive GO terms (Shared terms). The statistical significance of the overlap was determined by a one-tailed Fisher’s exact test.

To independently validate the core genes (Figure 2A) and functional modules (Figure 2D) identified in our broader comparative analysis (Methods), we utilized transcriptomic data from our previously generated autotetraploid plants of wild diploid potato [34]. Differential expression analysis revealed approximately 6000 DEGs in these potato autotetraploids, with their expression patterns summarized in a heatmap (Figure 4A). Our results showed a significant overlap (*p* = 7.1 × 10^−28^) between these potato DEGs and the 68 cross-species core DEGs (Figure 4B). Furthermore, GO enrichment analysis of the potato DEGs also revealed a significant overlap (*p* = 1.6 × 10^−10^) with the nine cross-species shared GO terms (Figure 4C).

Crucially, potato was not included in the initial discovery set for identifying core principles (Figure 1A). Nevertheless, these independent validation results from the autotetraploid potato model confirm the consistency of WGD-induced core genes and functional modules across diverse species. This newly presented potato data thus supports the biological patterns we identified.

## 4. Discussion

### 4.1. Intrinsic Stress Responses and Dosage Balance: Keys to Polyploid Establishment and Evolution

**Figure 5:**
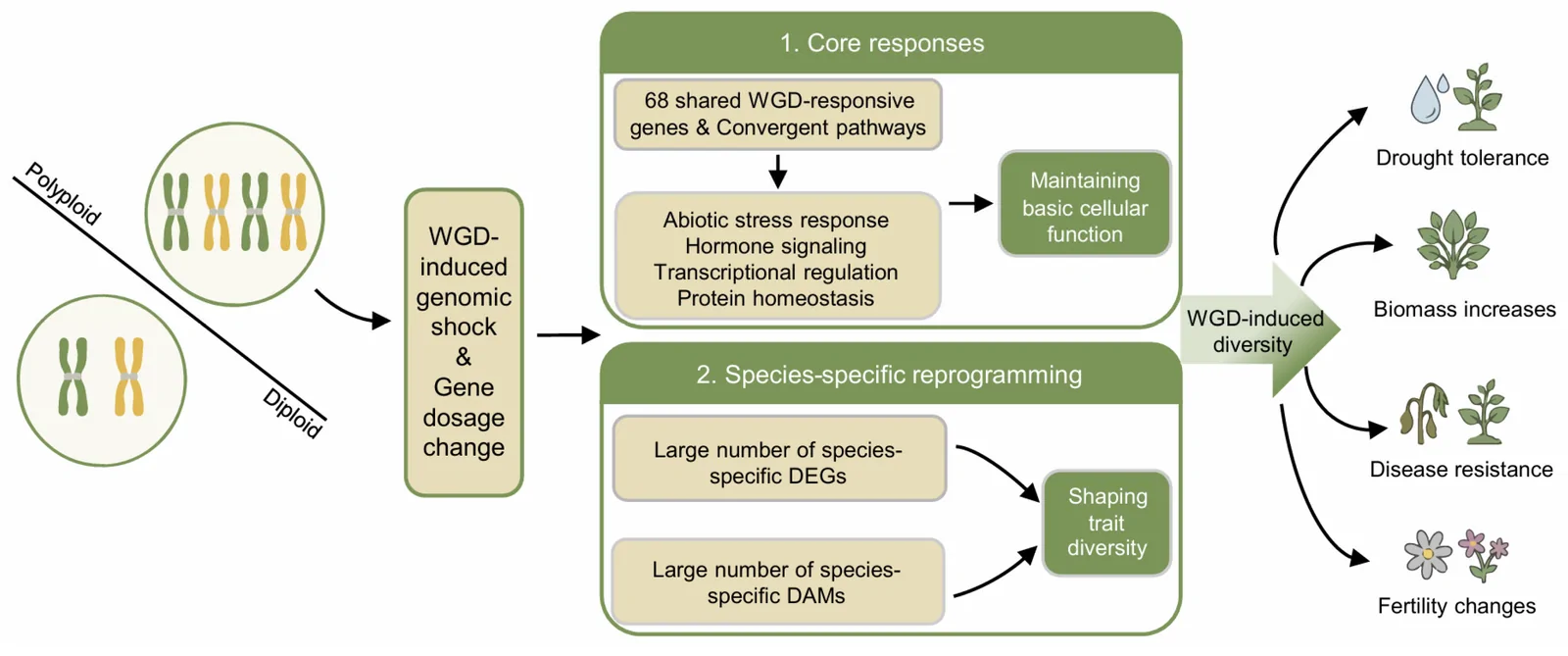
Conceptual model of WGD-induced molecular responses driving trait diversity. WGD initiates genomic shock and gene dosage change, leading to two parallel molecular responses: 1. Core responses involve 68 shared WGD-responsive genes and convergent pathways, crucial for maintaining basic cellular functions. 2. Species-specific reprogramming: driven by numerous species-specific DEGs and DAMs, shaping trait diversity. Together, these responses generate WGD-induced diversity, including enhanced drought tolerance, increased biomass, improved disease resistance, and altered fertility.

Accumulating evidence suggests that ancient WGD is strongly linked to lineage persistence and subsequent evolution, particularly during periods of environmental disruption [10,38,39]. Consistent with this view, our analysis of multi-species established WGD data revealed a core molecular response. Despite the absence of external stress, WGD consistently triggered significant functional enrichments in abiotic stress responses, hormone signaling, and homeostasis (Figure 5). This indicates that polyploidization itself initiates a robust transcriptomic and regulatory reorganization, mobilizing pre-existing functional networks typically deployed to manage environmental disturbance [25].

The 68 core WGD-responsive genes, validated in our independent autotetraploid potato model, comprise the types of elements expected to be crucial for buffering the genomic shock. These genes are often involved in transcriptional regulation (e.g., ERF, WRKY, and MYB), signaling pathways (e.g., CRK10, CML42, and IAA19), and protein homeostasis (e.g., ACD25.1, and HSFA2) (Supplementary Table S3), which are frequently dosage-sensitive components of complex regulatory networks or protein subunits. According to the gene balance hypothesis, maintaining precise dosage for such genes is critical, implying their coordinated regulation post-WGD is a fundamental survival mechanism [5,40]. This dosage-sensitive transcriptional and metabolic reprogramming profoundly influences the selective pressures that determine which duplicated genes are retained or lost during subsequent evolution [41]. Thus, these core responses not only ensure the maintenance of basic cellular function but also lay the groundwork for the genomic fate of polyploids.

Extending this perspective, we propose that the enrichment of ancient WGD events during periods of environmental disruption cannot be solely attributed to the genetic redundancy and evolutionary potential generated by genome doubling [6]. Instead, our study reflects the inherent propensity of WGD to activate these stress-response and homeostasis-related functional modules [25]. This intrinsic reprogramming provides an immediate physiological advantage, allowing newly formed polyploids to establish a more resilient state. By enhancing their likelihood of establishment and persistence under environmental selection pressures, WGD thus acts as a potent engine for plant diversification and adaptation [42,43].

### 4.2. Metabolite Specificity and Pathway Convergence Reveal a Hierarchical Organization of the WGD Response

In stark contrast to the transcriptome, where core DEGs were discernible, the metabolome exhibited no cross-species shared DAMs. This high species-specificity of individual metabolic changes is consistent with metabolites being more proximal to trait outputs, with their accumulation influenced by species-specific enzyme activity and substrate availability [44,45]. Consequently, metabolite alterations more readily reflect downstream trait divergence compared to gene expression responses [46].

However, despite this pronounced molecular divergence at the individual metabolite level, the DAM-enriched metabolic pathways demonstrated striking functional convergence (Figure 5). Collectively, these point to core physiological adjustment strategies deployed post-WGD, such as osmotic regulation, redox homeostasis, and membrane remodeling, centered on homeostatic reconfiguration and stress buffering [47,48]. From this perspective, our metabolomic results reveal a hierarchical organization of the WGD response across molecular layers, rather than simply mirroring transcriptomic enrichments [49]. Together, the transcriptomic and metabolomic findings underscore that cross-species commonality after WGD primarily resides not in specific individual genes or metabolites [50,51], but in the functional convergence towards common, fundamental physiological goals [47,48].

### 4.3. Core Responses and Species-Specific Reprogramming Jointly Shape the WGD-Induced Trait Diversity

Our identification of 68 orthologous response genes demonstrates that the molecular remodeling triggered by WGD adheres to a shared framework (Figure 5). This framework targets fundamental functions essential for environmental sensing, signal regulation, and expression program adjustment [49], directly addressing the universal challenges plants face post-ploidy transition. These challenges include altered gene dosage relationships, transcriptional networks, and metabolic resource allocation [40,52], necessitating the re-coordination of gene expression, protein folding, hormone balance, and overall homeostatic maintenance within the new ploidy context [25]. Consequently, a core set of modules is recurrently activated across lineages [37,53]. These often encompass upstream regulatory nodes that integrate stress adaptation and development [54], exemplified by IAA19—an Aux/IAA transcriptional repressor involved in drought adaptation and lateral root development [55], thus connecting environmental response and organ plasticity [56]. This shared cross-species response after WGD therefore represents a core regulatory framework integrating signal input and coordinating expression output [36].

In parallel with this shared core, species-specific transcriptional and metabolic reprogramming serves as a significant source of trait divergence after WGD [43]. This is evident at the transcriptional level through the substantial number of species-specific DEGs, and at the metabolic level by the absence of shared DAMs and the pronounced divergence of specific metabolic alterations. Due to inherent variations in life-history traits, genome structure, regulatory networks, and metabolic backgrounds, different plants channel the gene dosage changes and regulatory plasticity introduced by WGD into distinct directions. This ultimately leads to a spectrum of diverse outcomes, such as enhanced drought tolerance, biomass increases, disease resistance, or fertility changes [57]. Therefore, rather than enforcing identical changes, WGD safeguards basic cellular functions via a core response, while species-specific reprogramming shapes trait diversity (Figure 5).

### 4.4. Potential Applications of Core WGD-Responsive Genes

The 68 cross-species core WGD-responsive genes identified in this study constitute universal molecular signatures of polyploidization. Their consistent differential expression across our diverse multi-species WGD dataset positions them as a reliable tool for assessing polyploidization-associated responses. This enables the development of rapid molecular assays, particularly those based on gene-expression profiling (e.g., microarray or transcriptome analysis), which can complement traditional morphological observations and facilitate detection of ploidy transitions. When integrated with methods such as flow cytometry, chromosome counting, or genome-wide ploidy determination, these assays could enhance selection and material evaluation in polyploid breeding [58,59]. For widespread application and full validation, future research should systematically evaluate these core genes across diverse species, ploidy combinations, and developmental stages within a unified experimental framework.

## Data Availability

All data generated and analyzed in this study are provided in the Supplementary Data.

## Supplementary Materials

The following supporting information can be downloaded at: https://www.mdpi.com/article/10.3390/life16101643/s1.

## References

[1] Qiao X., Zhang S., Paterson A.H. (2022). Pervasive genome duplications across the plant tree of life and their links to major evolutionary innovations and transitions. Comput. Struct. Biotechnol. J..

[2] Jiao Y., Wickett N.J., Ayyampalayam S., Chanderbali A.S., Landherr L., Ralph P.E., Tomsho L.P., Hu Y., Liang H., Soltis P.S. (2011). Ancestral polyploidy in seed plants and angiosperms. Nature.

[3] Anderson A.W., Albertini E., Rosellini D. (2026). Polyploid plant genomes complexity and the challenges of sequencing. Planta.

[4] del Pozo J.C., Ramirez-Parra E. (2015). Whole genome duplications in plants: An overview from *Arabidopsis*. J. Exp. Bot..

[5] Birchler J.A., Veitia R.A. (2012). Gene balance hypothesis: Connecting issues of dosage sensitivity across biological disciplines. Proc. Natl. Acad. Sci. USA.

[6] Van de Peer Y., Mizrachi E., Marchal K. (2017). The evolutionary significance of polyploidy. Nat. Rev. Genet..

[7] Clark J.W., Donoghue P.C.J. (2018). Whole-Genome Duplication and Plant Macroevolution. Trends Plant Sci..

[8] Peeters M.K.R., Van de Peer Y. (2026). Polyploidy: A macromutational force pushing bioeconomic developments. Proc. Natl. Acad. Sci. USA.

[9] Bonte D., Van de Peer Y. (2026). A three-step model for the establishment of polyploid plants. Curr. Biol..

[10] Chen H., Almeida-Silva F., Logghe G., Maere S., Bonte D., Van de Peer Y. (2026). The rise of polyploids during environmental upheaval. Cell.

[11] Madadi M., Zhao K.L., Wang Y.M., Wang Y.T., Tang S.W., Xia T., Jin N.Z., Xu Z.J., Li G.H., Qi Z. (2021). Modified lignocellulose and rich starch for complete saccharification to maximize bioethanol in distinct polyploidy potato straw. Carbohydr. Polym..

[12] Yu Z., Haberer G., Matthes M., Rattei T., Mayer K.F.X., Gierl A., Torres-Ruiz R.A. (2010). Impact of natural genetic variation on the transcriptome of autotetraploid *Arabidopsis thaliana*. Proc. Natl. Acad. Sci. USA.

[13] Robinson D.O., Coate J.E., Singh A., Hong L., Bush M., Doyle J.J., Roeder A.H.K. (2018). Ploidy and size at multiple scales in the *Arabidopsis* sepal. Plant Cell.

[14] Lv P., Wang M., Qiu R., Yao C., Fang M., Xing Y., Zhang X., He Y., Cai D., Song Z. (2025). Comparative transcriptome analysis reveals key genes associated with meiotic stability and high seed setting rate in tetraploid rice. BMC Plant Biol..

[15] Zhang Y., Du A., Tong L., Yan G., Lu L., Yin Y., Fu X., Yang H., Li H., Huang W. (2024). Genome Resequencing for autotetraploid rice and its closest relatives reveals abundant variation and high potential in rice breeding. Int. J. Mol. Sci..

[16] Jiang J., Yang N., Li L., Qin G., Ren K., Wang H., Deng J., Ding D. (2022). Tetraploidy in *Citrus wilsonii* enhances drought tolerance via synergistic regulation of photosynthesis, phosphorylation, and hormonal changes. Front. Plant Sci..

[17] Soltis P.S., Soltis D.E. (2016). Ancient WGD events as drivers of key innovations in angiosperms. Curr. Opin. Plant Biol..

[18] Cai L., Xi Z., Amorim A.M., Sugumaran M., Rest J.S., Liu L., Davis C.C. (2019). Widespread ancient whole-genome duplications in *Malpighiales coincide* with Eocene global climatic upheaval. New Phytol..

[19] Sessa E.B. (2019). Polyploidy as a mechanism for surviving global change. New Phytol..

[20] Tasdighian S., Sensalari C., Maere S. (2026). Using reciprocally retained gene families to detect whole-genome multiplications in plants. Genome Biol..

[21] Shi T., Van de Peer Y. (2026). Revisiting ancient whole-genome duplications in the seed and flowering plants through the lens of dosage-sensitive genes. Sci. Adv..

[22] Wendel J.F. (2015). The wondrous cycles of polyploidy in plants. Am. J. Bot..

[23] Edger P.P., Soltis D.E., Yoshioka S., Vallejo-Marin M., Shimizu-Inatsugi R., Shimizu K.K., Salmon A., Hiscock S., Ainouche M., Soltis P.S. (2025). Natural neopolyploids: A stimulus for novel research. New Phytol..

[24] Machlańska A., Marasek-Ciołakowska A., Podwyszyńska M., Pluta S. (2026). The impact of genome duplication on agronomically valuable traits in *Ribes nigrum* L.: A morpho-anatomical, physiological and cytogenetic evaluation. BMC Plant Biol..

[25] Bomblies K. (2020). When everything changes at once: Finding a new normal after genome duplication. Proc. R. Soc. B Biol. Sci..

[26] Fasano C., Diretto G., Aversano R., D’Agostino N., Di Matteo A., Frusciante L., Giuliano G., Carputo D. (2016). Transcriptome and metabolome of synthetic Solanum autotetraploids reveal key genomic stress events following polyploidization. New Phytol..

[27] Jin Y., Qian H.V. (2022). PhyloMaker2: An updated and enlarged R package that can generate very large phylogenies for vascular plants. Plant Divers..

[28] Xie J.M., Chen Y.R., Cai G.J., Cai R.L., Hu Z., Wang H. (2023). Tree Visualization By One Table (tvBOT): A web application for visualizing, modifying and annotating phylogenetic trees. Nucleic Acids Res..

[29] Chen S., Zhou Y., Chen Y., Gu J. (2018). fastp: An ultra-fast all-in-one FASTQ preprocessor. Bioinformatics.

[30] Patro R., Duggal G., Love M.I., Irizarry R.A., Kingsford C. (2017). Salmon provides fast and bias-aware quantification of transcript expression. Nat. Methods.

[31] Love M.I., Huber W., Anders S. (2014). Moderated estimation of fold change and dispersion for RNA-seq data with DESeq2. Genome Biol..

[32] Emms D.M., Kelly S. (2019). OrthoFinder: Phylogenetic orthology inference for comparative genomics. Genome Biol..

[33] Chen C.J., Wu Y., Li J.W., Wang X., Zeng Z.H., Xu J., Liu Y.L., Feng J.T., Chen H., He Y.H. (2023). TBtools-II: A “one for all, all for one” bioinformatics platform for biological big-data mining. Mol. Plant.

[34] Zhao K., Jin N., Madadi M., Wang Y., Wu L., Xu Z., Wang J., Dong J., Tang S.W., Wang Y. (2022). Incomplete genome doubling enables to consistently enhance plant growth for maximum biomass production by altering multiple transcript co-expression networks in potato. Theor. Appl. Genet..

[35] Banouh M., Armisen D., Bouguennec A., Huneau C., Sow M.D., Pont C., Salse J., Civáň P. (2023). Low impact of polyploidization on the transcriptome of synthetic allohexaploid wheat. BMC Genom..

[36] Gillard G.B., Grønvold L., Røsæg L.L., Holen M.M., Monsen Ø., Koop B.F., Rondeau E.B., Gundappa M.K., Mendoza J., Macqueen D.J. (2021). Comparative regulomics supports pervasive selection on gene dosage following whole genome duplication. Genome Biol..

[37] Jackson S., Chen Z.J. (2010). Genomic and expression plasticity of polyploidy. Curr. Opin. Plant Biol..

[38] Fawcett J.A., Maere S., Van de Peer Y. (2009). Plants with double genomes might have had a better chance to survive the Cretaceous–Tertiary extinction event. Proc. Natl. Acad. Sci. USA.

[39] Vanneste K., Baele G., Maere S., Van de Peer Y. (2014). Analysis of 41 plant genomes supports a wave of successful genome duplications in association with the Cretaceous–Paleogene boundary. Genome Res..

[40] Birchler J.A., Veitia R.A. (2007). The gene balance hypothesis: From classical genetics to modern genomics. Plant Cell.

[41] Song M.J., Potter B.I., Doyle J.J., Coate J.E. (2020). Gene balance predicts transcriptional responses immediately following ploidy change in *Arabidopsis thaliana*. Plant Cell.

[42] Comai L. (2005). The advantages and disadvantages of being polyploid. Nat. Rev. Genet..

[43] Van de Peer Y., Ashman T.-L., Soltis P.S., Soltis D.E. (2021). Polyploidy: An evolutionary and ecological force in stressful times. Plant Cell.

[44] Fiehn O. (2002). Metabolomics—The link between genotypes and phenotypes. Plant Mol. Biol..

[45] Ramon C., Stelling J. (2023). Functional comparison of metabolic networks across species. Nat. Commun..

[46] Lai W.-Y., Otte K.A., Schlötterer C. (2023). Evolution of metabolome and transcriptome supports a hierarchical organization of adaptive traits. Genome Biol. Evol..

[47] Krasensky J., Jonak C. (2012). Drought, salt, and temperature stress-induced metabolic rearrangements and regulatory networks. J. Exp. Bot..

[48] Foyer C.H., Noctor G. (2005). Redox homeostasis and antioxidant signaling: A metabolic interface between stress perception and physiological responses. Plant Cell.

[49] Sobhanian H., Song W.-Y., Soltis P.S., Soltis D.E., Chen S. (2026). Polyploidy and plant resilience to environmental stresses: Molecular mechanisms and future applications. Plant Commun..

[50] Baharum S.N., Azizan K.A., Aizat W.M., Goh H.-H., Baharum S.N. (2018). Metabolomics in Systems Biology. Omics Applications for Systems Biology.

[51] Bohutínská M., Alston M., Monnahan P., Mandáková T., Bray S., Paajanen P.A.-O., Kolář F., Yant L. (2021). Novelty and convergence in adaptation to whole genome duplication. Mol. Biol. Evol..

[52] Bekaert M., Edger P.P., Pires J.C., Conant G.C. (2011). Two-phase resolution of polyploidy in the *Arabidopsis* metabolic network gives rise to relative and absolute dosage constraints. Plant Cell.

[53] Scarrow M., Wang Y., Sun G. (2021). Molecular regulatory mechanisms underlying the adaptability of polyploid plants. Biol. Rev..

[54] Edger P.P., Body M.J.A., De Donno S., Platts A.E., Wang J., Jiang J. (2026). Genome evolution through polyploidy: Enhancing plant stress resilience in agriculture. Proc. Natl. Acad. Sci. USA.

[55] Salehin M., Li B., Tang M., Katz E., Song L., Ecker J.R., Kliebenstein D.J., Estelle M. (2019). Auxin-sensitive Aux/IAA proteins mediate drought tolerance in *Arabidopsis* by regulating glucosinolate levels. Nat. Commun..

[56] Shani E., Salehin M., Zhang Y., Sanchez S.E., Doherty C., Wang R., Mangado C.C., Song L., Tal I., Pisanty O. (2017). Plant stress tolerance requires auxin-sensitive Aux/IAA transcriptional repressors. Curr. Biol..

[57] Sattler M.C., Carvalho C.R., Clarindo W.R. (2016). The polyploidy and its key role in plant breeding. Planta.

[58] Zhao K.L., Dong J., Xu J.X., Bai Y.B., Yin Y.H., Long C.S., Wu L., Lin T.R., Fan L.Q., Wang Y.F. (2024). Downregulation of the expression of subgenomic chromosome A7 genes promotes plant height in resynthesized allopolyploid *Brassica napus*. Theor. Appl. Genet..

[59] Zhao K.L., Bai Y.B., Zhang Q.Y., Zhao Z., Cao Y., Yang L., Wang N., Xu J.X., Wang B., Wu L. (2023). Karyotyping of aneuploid and polyploid plants from low coverage whole-genome resequencing. BMC Plant Biol..

